# Whole Exome Sequencing Is the Preferred Strategy to Identify the Genetic Defect in Patients With a Probable or Possible Mitochondrial Cause

**DOI:** 10.3389/fgene.2018.00400

**Published:** 2018-10-12

**Authors:** Tom E. J. Theunissen, Minh Nguyen, Rick Kamps, Alexandra T. Hendrickx, Suzanne C. E. H. Sallevelt, Ralph W. H. Gottschalk, Chantal M. Calis, Alphons P. M. Stassen, Bart de Koning, Elvira N. M. Mulder-Den Hartog, Kees Schoonderwoerd, Sabine A. Fuchs, Yvonne Hilhorst-Hofstee, Marianne de Visser, Jo Vanoevelen, Radek Szklarczyk, Mike Gerards, Irenaeus F. M. de Coo, Debby M. E. I. Hellebrekers, Hubert J. M. Smeets

**Affiliations:** ^1^Department of Genetics and Cell Biology, Maastricht University Medical Centre, Maastricht, Netherlands; ^2^Research Institute GROW, Maastricht University Medical Centre, Maastricht, Netherlands; ^3^Department of Pediatric Neurology, Erasmus MC Sophia Children's Hospital, Rotterdam, Netherlands; ^4^Department of Clinical Genetics, Erasmus MC, Rotterdam, Netherlands; ^5^Department of Metabolic Disorders, University Medical Centre Utrecht, Utrecht, Netherlands; ^6^Department of Clinical Genetics, Leiden University Medical Centre, Leiden, Netherlands; ^7^Department of Neurology, Academic Medical Centre Amsterdam, Amsterdam, Netherlands; ^8^Maastricht Center for Systems Biology (MaCSBio), Maastricht University Medical Centre, Maastricht, Netherlands

**Keywords:** mitochondrial disease, next-generation sequencing, mtDNA sequencing, whole-exome sequencing, diagnostic yield

## Abstract

Mitochondrial disorders, characterized by clinical symptoms and/or OXPHOS deficiencies, are caused by pathogenic variants in mitochondrial genes. However, pathogenic variants in some of these genes can lead to clinical manifestations which overlap with other neuromuscular diseases, which can be caused by pathogenic variants in non-mitochondrial genes as well. Mitochondrial pathogenic variants can be found in the mitochondrial DNA (mtDNA) or in any of the 1,500 nuclear genes with a mitochondrial function. We have performed a two-step next-generation sequencing approach in a cohort of 117 patients, mostly children, in whom a mitochondrial disease-cause could likely or possibly explain the phenotype. A total of 86 patients had a mitochondrial disorder, according to established clinical and biochemical criteria. The other 31 patients had neuromuscular symptoms, where in a minority a mitochondrial genetic cause is present, but a non-mitochondrial genetic cause is more likely. All patients were screened for pathogenic variants in the mtDNA and, if excluded, analyzed by whole exome sequencing (WES). Variants were filtered for being pathogenic and compatible with an autosomal or X-linked recessive mode of inheritance in families with multiple affected siblings and/or consanguineous parents. Non-consanguineous families with a single patient were additionally screened for autosomal and X-linked dominant mutations in a predefined gene-set. We identified causative pathogenic variants in the mtDNA in 20% of the patient-cohort, and in nuclear genes in 49%, implying an overall yield of 68%. We identified pathogenic variants in mitochondrial and non-mitochondrial genes in both groups with, obviously, a higher number of mitochondrial genes affected in mitochondrial disease patients. Furthermore, we show that 31% of the disease-causing genes in the mitochondrial patient group were not included in the MitoCarta database, and therefore would have been missed with MitoCarta based gene-panels. We conclude that WES is preferable to panel-based approaches for both groups of patients, as the mitochondrial gene-list is not complete and mitochondrial symptoms can be secondary. Also, clinically and genetically heterogeneous disorders would require sequential use of multiple different gene panels. We conclude that WES is a comprehensive and unbiased approach to establish a genetic diagnosis in these patients, able to resolve multi-genic disease-causes.

## Introduction

Mitochondrial disorders are clinically highly heterogeneous, with a broad variety of neurological and muscular symptoms involved and having significant clinical overlap with other neuromuscular disorders. Although, mitochondrial disorders are characterized by deficiencies in the oxidative phosphorylation (OXPHOS) and ATP production, biochemical deficiencies are not always detected in the lab. Besides, OXPHOS deficiencies can be a secondary phenomenon in neuromuscular or multi-system disorders with a non-mitochondrial cause (Pyle et al., 2015; Niyazov et al., 2016). Mitochondrial disorders are also genetically heterogeneous, as different gene defects can result in a similar phenotype and both the nuclear and mitochondrial genomes are involved (Rotig and Munnich, 2003; McFarland et al., 2010). These features complicate the establishment of a genetic diagnosis in mitochondrial patients (Koenig, 2008).

For pathogenic variants in the multi-copy mtDNA, which contains 37 genes and is exclusively maternally inherited, the mutation load of the so-called heteroplasmic pathogenic variants also affects the clinical presentation (Thorburn and Dahl, 2001; Hellebrekers et al., 2012). The estimated number of nuclear genes involved in mitochondrial function is around 1500 (Prokisch and Ahting, 2007; Calvo and Mootha, 2010), of which only > 250 genes have been shown to be involved in mitochondrial disease (Alston et al., 2017). In mitochondrial disorders (patients in group 1), OXPHOS deficiencies are often due to genetic defects in the OXPHOS complexes (subunits and assembly factors), or, more indirectly, in processes such as mitochondrial protein translation and degradation, mtDNA maintenance, fusion and fission, substrate transport, or phospholipid metabolism. Still, mitochondrial dysfunction can also be a more secondary defect in genetic syndromes or neuromuscular disease (Hui et al., 2006). Also, in patients with neuromuscular symptoms that are not specific for a mitochondrial disease (patients in group 2), pathological variants in mitochondrial genes have been reported in addition to other non-mitochondrial genetic causes.

Genetic diagnosis of mtDNA-disorders requires screening of all 37 mtDNA genes and determining the heteroplasmy levels of the variants, being either point mutations or large rearrangements. In addition, mtDNA copy-number is being tested to identify mtDNA depletions. mtDNA analysis in diagnostic setting commonly started with screening for a few relatively common point mutations using mutation-specific restriction enzymes or qPCR based methods (Fan et al., 2006; Wang et al., 2011). In case common pathogenic variants were not detected, the entire mitochondrial genome was analyzed by Sanger sequencing or chip-based methods (van Eijsden et al., 2006; Finsterer et al., 2009). However, these methods are non-quantitative, requiring a second molecular test to determine the mutation load. Besides, chip-based methods have difficulties in detecting small indels (Tang and Huang, 2010; Xie et al., 2011; McCormick et al., 2013). For the large mtDNA deletions, it was time-consuming to determine the exact breakpoints, which could be important for prognosis (He et al., 2002; Wong et al., 2003). The application of next-generation sequencing has greatly increased the possibilities for detecting, characterizing and quantifying point mutations, and rearrangements across the complete mitochondrial genome with one single technology (Li et al., 2010; Huang, 2011; Cui et al., 2013). Although, accurate determination of the heteroplasmy levels of large mtDNA deletions still requires quantitative PCR analysis. The first tissue tested is blood, but this is extended to muscle or urine in case the mtDNA could have been missed in blood (Koenig, 2008). If no pathogenic variants in the mtDNA were present, then moving into the analysis of nuclear genes traditionally relied on (stepwise) Sanger sequencing of nuclear candidate genes which were selected based on clinical and biochemical features or linkage/homozygosity mapping. Again next-generation sequencing methods have made this approach obsolete, as whole exome sequencing (WES), enabled the detection of the majority of the genetic variations in the coding part of the genome (Wortmann et al., 2015). An unbiased and complete genetic analysis is important, especially in heterogeneous mitochondrial disease, where genotype-phenotype relations can be indistinct and novel genes involved.

We have performed a complete next-generation sequencing strategy, analysing the mtDNA and exome in a cohort of 86 patients with mitochondrial disease (group 1) and 31 neuromuscular patients in whom a pathogenic variant in a mitochondrial gene could possibly be involved (group 2). The latter group includes heterogeneous neuromuscular patients with disease symptoms that are not specific for mitochondrial disease, but in which a mitochondrial genetic cause has been identified in a minority of cases. The results were obtained over a 4-year period from 2012 to 2016. Patients were tested for pathogenic variants in the mtDNA by next-generation sequencing and, if negative, further analyzed by WES. WES-variants were filtered according to the presumed genetic model of disease inheritance, allele frequencies, conservation, and the predicted effect of the variant. With this approach, we identified in 68% of our patients a causative pathogenic variant in new and known disease genes, which were either inherited or *de novo*.

## Material and methods

### Patients

One hundred and seventeen patients, from consanguineous and non-consanguineous families, in this study were under treatment at Erasmus MC, Maastricht UMC+, Leiden UMC, UMC Utrecht, AMC Amsterdam (The Netherlands). The patients had a clinical phenotype that could be caused by a mitochondrial defect. The age of disease onset in the cohort varied from birth to approximately 50 years old. As 77% of our patients was below 18 years at the age of diagnosis we used the mitochondrial disease criteria (MDC) for children (Morava et al., 2006). The MDC include clinical signs and symptoms (max 4 points), metabolic abnormalities and neuroimaging (max 4 points) and histologic anomalies (max 4 points). Score 1: mitochondrial disorder unlikely; score 2 to 4: possible mitochondrial disorder; score 5 to 7: probable mitochondrial disorder; score 8 to 12: definite mitochondrial disorder. In group I patients were included, in whom the diagnosis of a mitochondrial disease was probable or definite (MDC > 5). Patients not meeting these MD-criteria (MDC < 5, group 2) had neuromuscular symptoms that are not specific for mitochondrial disease, but in which a mitochondrial genetic cause has been identified in a minority of cases. All parents were unaffected. Subjects gave written informed consent for WES analysis in accordance with the Declaration of Helsinki. Research was prospectively reviewed and approved by the local ethical committee of the Maastricht University Medical Centre.

### mtDNA analysis

Sequencing of the mtDNA was performed using the Illumina MiSeq platform. Enrichment of the entire mtDNA was performed by a single long-range PCR using Phusion Hot Start DNA polymerase II kit (Thermoscientific) and 100 ng of total genomic DNA, according to the manufacturer's instructions. Library preparation was performed by the Illumina Nextera XT kit according to the manufacturer's instructions. Twelve indexed DNA libraries were equimolarly pooled and sequenced in a single lane of 1 MiSeq flow-cell with a 2 × 300 bp paired-end chemistry. Data demultiplexing was performed with Illumina CASAVA software (v.1.8.4.) and reads were aligned against the revised Cambridge mitochondrial reference sequence (but without the gap at position 3107) by BWA software (v.0.5.9.) (Li and Durbin, 2009). For both variant and small indel identification, Python 2.6.6., Python package pysam 0.7.8. and SAMTools 0.1.19 software were used (Li et al., 2009). Large deletions were identified by alignment with the Smith and Waterman algorithm and the EMBOSS water program (v.6.5.7.). Annotation and filtering of mtDNA variants and indels were performed using in-house build Perl tools and a MySQL annotation database. Calculation of the heteroplasmy level at any nucleotide position was performed by the read depth of the mutant vs. reference nucleotide. We have validated the detection and quantification accuracy of our NGS strategy by analysing DNA samples with varying heteroplasmy levels of different substitution variants, small indels and large deletions, as previously determined by MitoChip, RFLP and PCR/Southern blotting (Supplementary Data Tables S1–S5; SRA submission SUB4444333). The entire mtDNA, excluding the highly polymorphic D-loop, was analyzed using a 2% heteroplasmy cut-off for known pathogenic point mutations and a 5% cut-off for the remaining positions and small indels. If no mtDNA mutations were detected in blood of the MD-patient (group 1) and a mtDNA disease was highly likely, this was confirmed on muscle or urine DNA. To test for mtDNA depletions, qPCR quantification was performed on DNA extracted from available muscle biopsies, using SensiMix SYBR (Bioline), where mtDNA copy-number (based on the mitochondrial ND1 gene) was normalized to the nDNA copy-number (based on the nuclear B2M gene).

### Homozygosity mapping and WES

Homozygosity mapping and CNV analysis was performed by HumanMapping 250K array (Affymetrix, Santa Clara, California) and Genotyping console 4.0 (Affymetrix). Homozygosity or hemizygosity regions were defined by the “Homozygosity” mapper (Seelow et al., 2009), with a cutoff of 5 MB. Exons were captured with SureSelect version 5 exome enrichment kit (Agilent Technologies, Santa Clara, California), including untranslated regions. Sequencing was performed on a HiSeq2000 platform (Illumina, San Diego, California), using a 2 × 100 bp paired-end setting. Bcl2fastq 1.8.4 (Illumina) allowed Basecalling and demultiplexing, and Burrows-Wheeler Aligner 0.5.9 (Broad Institute, Cambridge, Massachusetts) was used for read alignment against human reference genome hg19. Duplicate reads were removed by Picard software suite 1.77 (Broad Institute, Cambridge, Massachusetts) and variant calling was performed with GATK 2.1-8 (Broad Institute).

Exome data of consanguineous families or families consisting of > 1 patient was filtered for recessive homozygous, compound heterozygous, and X-linked (XLR) pathogenic variants. Variants with allele frequencies <1% (dbSNP151 Exome Aggregation Consortium) were evaluated, covering missense variants, indels, nonsense mutations, and splice variants. Non-annotated variants were maintained, unless allele frequencies exceeded 5% prevalence in our in-house patient database. Pathogenicity of nonsynonymous missense variants was estimated by Polymorphism Phenotyping-2 (PolyPhen-2; Harvard, Boston, Massachusetts), Sorting Intolerant From Tolerant (J. Craig Venter Institute, Rockville, Maryland), Protein Variation Effect Analyzer (PROVEAN; J. Craig Venter Institute), and MutationTaster (NeuroCure Cluster of Excellence/Berlin Institute of Health, Berlin, Germany). Nonsense, frameshift, and splice-site variations were maintained. WES data of single patients from non-consanguineous families were additionally filtered for heterozygous variants in known (OMIM (Online Mendelian Inheritance in Man) disease genes with known dominant and/or *de novo* pathogenic variants. All identified pathogenic variants were checked for their inclusion in the MitoCarta build 2.0 database for mitochondrial localized proteins.

All variants that are listed in this manuscript were assigned as “most probably disease causing” (class 4) or “disease causing” (class 5). “Disease causing” (class 5), based on previous publications reporting patients with a similar phenotype, possibly with functional prove, or, in case of unpublished variants, classified as “most probably disease causing” (class 4), according to the diagnostic standards and guidelines of the American College of Medical Genetics and Genomics (ACMG) (Richards et al., 2015). The ACMG criteria take into account, among others: allele frequencies in control databases (missense variants), amino-acid conservation, Grantham score, functional domains, and splice predictions and nucleotide conservation in case of intronic variants. Pathogenic variants were only included if these segregated correctly with the disease in the family.

## Results

### Diagnostic yield

**Figure 1:**
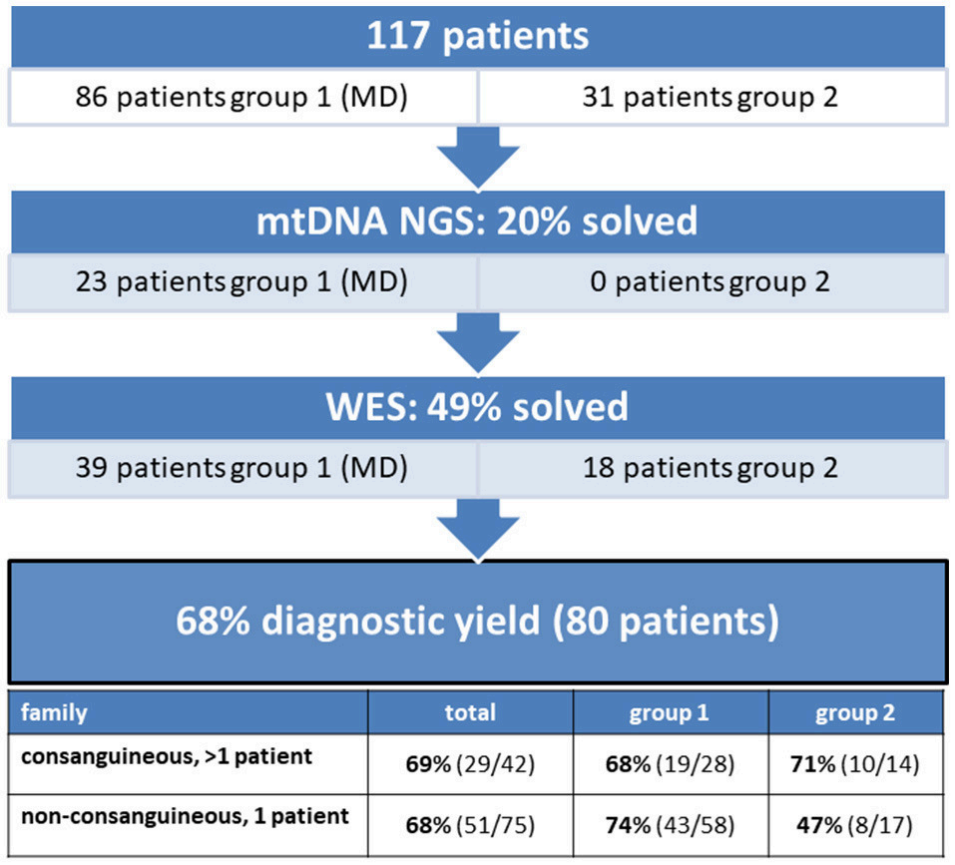
The diagnostic yield of mtDNA and whole exome sequencing in a patient cohort consisting of 117 patients. 20% of the patients were solved with a mtDNA defect and 49% with a nuclear DNA defect, implying an overall diagnostic yield of 68%.

A cohort of 117 patients from unaffected parents, was subject to a two-step next-generation sequencing approach (Figure 1). The age of disease onset in the cohort varied from birth to approximately 50 years old, 77% (90/117) were patients <18 years of age. We solved 20% of the cohort with a disease-causing mtDNA defect, involving 23 MD-patients from group 1 and none from group 2. Subsequent WES analysis solved an additional 49% (57 patients) of the cohort, consisting of 39 patients of group 1 and 18 patients from group 2. With this strategy, we achieved an overall diagnostic yield of 68%, with comparable yield in the group of inherited disease cases (69%) compared to the single patients from non-consanguineous families (68%).

### mtDNA next-generation sequencing

mtDNA sequencing resulted in the identification of a disease-causing pathogenic variants in 20% (23/117) of the patient cohort (Figure 1), solving 5% (2/42) of the inherited disease cases (consanguineous, > 1 patient) and 28% (21/75) of the single patients from non-consanguineous families. As shown in Table 1, all cases with a mtDNA defect were MD-patients (group 1) and all 23 identified mtDNA defects involved known mtDNA mutations in typical mtDNA disorders, like LHON (Leber's hereditary optic neuropathy), MELAS (mitochondrial encephalomyopathy, lactic acidosis, and stroke-like episodes)/MIDD (maternally inherited diabetes mellitus and deafness), Leigh syndrome and CPEO (chronic progressive external ophthalmoplegia)/Kearns-Sayre syndrome. 39% (9/23) of the mtDNA mutations were, mostly homoplasmic (8 out of 9), LHON mutations (m.11778G>A and m.14484T>C), clinically manifesting with optic atrophy. Only one LHON patient carried 80% heteroplasmy. The m.3243A>G mutation covered 30% (7/23) of the mtDNA defects, and explained both MELAS (2 patients) and MIDD (5 patients) phenotypes. Another mutation was identified in the ND5 gene (m.13513G>A), as a cause of Leigh syndrome. 26% (6/23) of the mtDNA defects were large single deletions, of which the 4,977 bp deletion (breakpoints 8482:13460) was detected in 4 patients. These patients had CPEO, sometimes in combination with additional symptoms of Kearns-Sayre syndrome (pigmentary retinopathy, cardiac conduction abnormalities). Patients with multiple mtDNA deletions or mtDNA depletion (not included in Table 1) were further analyzed by WES, as being suggestive of an underlying nuclear DNA defect in the mtDNA maintanance or replication genes.

**Table 1:** Disease-causing pathogenic variants identified by mtDNA sequencing.

		**Family**	**Patients**	**Pediatric/adult**	**Symptoms**	**Mutation**	**% Mutant mtDNA (in blood)**	**Gene**
Cons., >1 patient	MD	Non-consanguineous	2	a	LHON	m.11778G>A	Homoplasmic	*MT-ND4*
		Non-consanguineous	2	a	LHON	m.11778G>A	Homoplasmic	*MT-ND4*
Non-consanguineous, 1 patient	MD	Non-consanguineous	1	a	MELAS	m.3243A>G	24% (32 y.o.)	*MT-TL1*
		Non-consanguineous	1	p	MELAS	m.3243A>G	41% (18 y.o.)	*MT-TL1*
		Non-consanguineous	1	a	MIDD, ptosis, macular degeneration	m.3243A>G	25% (36 y.o.)	*MT-TL1*
		Non-consanguineous	1	a	MIDD	m.3243A>G	26% (27 y.o)	*MT-TL1*
		Non-consanguineous	1	a	MIDD	m.3243A>G	10% (62 y.o.)	*MT-TL1*
		Non-consanguineous	1	a	MIDD, macular degeneration	m.3243A>G	7% (69 y.o.)	*MT-TL1*
		Non-consanguineous	1	a	MIDD, cardiomyopathy	m.3243A>G	40% (28 y.o.)	*MT-TL1*
		Non-consanguineous	1	p	Leigh syndrome	m.13513G>A	72%	*MT-ND5*
		Non-consanguineous	1	a	LHON	m.11778G>A	80%	*MT-ND4*
		Non-consanguineous	1	a	LHON	m.11778G>A	Homoplasmic	*MT-ND4*
		Non-consanguineous	1	a	LHON	m.11778G>A	Homoplasmic	*MT-ND4*
		Non-consanguineous	1	a	LHON	m.11778G>A	Homoplasmic	*MT-ND4*
		Non-consanguineous	1	a	LHON	m.11778G>A	Homoplasmic	*MT-ND4*
		Non-consanguineous	1	a	LHON	m.14484T>C	Homoplasmic	*MT-ND6*
		Non-consanguineous	1	a	LHON	m.14484T>C	Homoplasmic	*MT-ND6*
		Non-consanguineous	1	p	CPEO	*de novo*: single 8284 bp deletion (7462:15747)	n.d.	covers 22 mtDNA genes
		Non-consanguineous	1	p	CPEO	*de novo*: single 6277 bp deletion (9514-15792)	n.d.	*MT-CO3, MT-ND3, MT-ND4L, MT-ND4, MT-ND5, MT-ND6, MT-CYB*, tRNA's (Leu, Ser, His, Gly, Arg, Glu)
		Non-consanguineous	1	p	CPEO	*de novo*: single 4977 bp deletion (breakpoints 8482:13460)	n.d.	*MT-ATP8, MT-ATP6, MT-CO3, MT-ND3, MT-ND4L, MT-ND4, MT-ND5*, tRNA's (Leu, Ser, His, Gly, Arg)
		Non-consanguineous	1	p	CPEO	*de novo*: single 4977 bp deletion (breakpoints 8482:13460)	n.d.	*MT-ATP8, MT-ATP6, MT-CO3, MT-ND3, MT-ND4L, MT-ND4, MT-ND5*, tRNA's (Leu, Ser, His, Gly, Arg)
		Non-consanguineous	1	p	CPEO	*de novo*: single 4,977 bp deletion (breakpoints 8482:13460)	n.d.	*MT-ATP8, MT-ATP6, MT-CO3, MT-ND3, MT-ND4L, MT-ND4, MT-ND5*, tRNA's (Leu, Ser, His, Gly, Arg)
		Non-consanguineous	1	p	Kearns-Sayre syndrome	*de novo*: single 4977 bp deletion (breakpoints 8482:13460)	n.d.	*MT-ATP8, MT-ATP6, MT-CO3, MT-ND3, MT-ND4L, MT-ND4, MT-ND5*, tRNA's (Leu, Ser, His, Gly, Arg)

*mtDNA point mutations and deletions were detected and quantified by mtDNA next-generation sequencing in a cohort of 117 patients. All patients with a mtDNA defect were classified as mitochondrial (group 1)*.

### Whole exome sequencing

In the remaining 94 patients, prior to whole exome analysis, the DNA were subject to SNP-array analysis, using the Affymetrix GeneChip Human Mapping 250K, to detect copy number variations (CNV) and homozygosity regions. No CNVs were detected. In patients from consanguineous families, mapping of the homozygous regions provided an additional filtering criterion to select the most promising genetic variants from the exome data. When multiple affected siblings were present, the entire family, if available, was analyzed by means of SNP-arrays. This approach significantly increased the efficiency of interpreting WES results. WES identified a disease-causing gene defect in 49% (57/117) of the complete patient cohort (Figure 1). In an additional 7% of the cohort, WES analysis identified a genetic variant which could explain the patient's phenotype, but for which definite evidence is currently lacking. Further studies should reveal which of these pathogenic variants could be classified as disease-causing.

WES-data filtering was based on the presumed genetic mode of inheritance. We applied a variant selection strategy for autosomal recessive (AR) and recessive X-linked (XLR) disorders to the inherited disease cases (consanguinity, >1 patient). As shown in Table 2, this group consisted of 40 families, where WES identified of a genetic defect in 68% (27/40) of the cases, covering homozygous, compound heterozygous and X-linked pathogenic variants (Tables 3, 4). In group 1 (MD patients), 65% (17/26) of the cases were solved, and in group 2 a disease causing gene defect was found in 71% (10/14). As expected, a broad spectrum of genes with relatively few genes with a literature reported mitochondrial function [20% (2/10)] was identified in group 2 (Table 4) compared to the proportion of such genes in group 1 [76% (13/17)] (Table 3). In 54 non-consanguineous families with a single patient, we applied a variant selection strategy for autosomal recessive (AR), X-linked (XLR and XLD) and single heterozygous pathogenic variants, expected to be dominant *de novo* variants. In order to limit the huge amount of heterozygous variants for dominant disease-causing mutations, variant interpretation was restricted to genes that have previously been reported in dominant disease based on the OMIM database. With this approach, we unraveled the genetic cause in 56% (30/54) of these families (Table 2), 15% of which were single dominant pathogenic variants. Follow-up investigations in the parents revealed that the pathogenic variants either occurred *de novo* in the patients or were present in one of the parent, who displayed subclinical symptoms upon further investigation. The diagnostic yield was 59% (22/37) in patient group 1 and 47% (8/17) in group 2, where respectively, 86% (19/22) (Table 3) and 13% (1/8) (Table 4) of the defects were located in a gene with reported mitochondrial function.

**Table 2:** Whole-exome sequencing analysis.

**WES filtering strategy**	**Total % solved**	**Group 1 % solved**	**Group 2 % solved**
Consanguineous and/or >1 patient Autosomal recessive (AR) X-linked (XLR)	68% (27/40)	65% (17/26)	71% (10/14)
Non-consanguineous, 1 patient Autosomal recessive (AR) X-linked (XLR and XLD) Autosomal dominant (AD)	56% (30/54) of which 15% *de novo* (8/54)	59% (22/37)	47% (8/17)

*94 patients were subject to WES analysis. WES-data was filtered according to the presumed pattern of disease inheritance, where consanguinity or involvement of >1 patient were indicative of a recessively inherited disorder and non-consanguineous families with a single patient were likely to cover both, recessively inherited and dominant de novo mutations*.

**Table 3:** Disease-causing pathogenic variants identified by WES in group 1, consisting of mitochondrial patients (MD).

	**Family**	**Patients**	**Pediatric/ adult**	**Symptoms (key features)**	**Biochemistry (muscle or fibroblasts)**	**MDC Score**	**Orientation**	**Inheritance**	**Mito Carta**	**Gene (published by our group)**	**Pathogenic variant**
**Autosomal recessive, X-linked (consanguineous**, >**1 patient)**	Non-consanguineous	3	p	Mitochondrial myopathy, hypertrophic cardiomyopathy	Combined OXPHOS deficiency	8	Compound heterozygous	AR	Yes	*AARS2* (Kamps et al., 2018)	c.1774G>A, p.(Gly85Arg); c.938G>A, p.(Arg958*)
	Consanguineous	2 (partial overlap)	p	Multi-system, Leigh-like, encephalopathy, 3-methylglutaconic aciduria, aminoacylase 1 deficiency, skin lesions	Normal	8	Homozygous, homozygous, homozygous	AR, AR, AR	Yes, no, no	*SERAC1, ACY1, ANTXR2* (Theunissen et al., 2017b)	c.1347_1349dup, p.(Ser450dup); c.811G>A, p.(Ala271Thr); c.1142A>G, p.(Tyr381Cys)
	Consanguineous	2	p	Encephalomyopathy, cerebellar atrophy, muscle weakness	CI deficiency	6	Homozygous	AR	Yes	*SPG7*	c.861dup, p.(Asn288*)
	Consanguineous	2	p	Leigh-like, mitochondrial encephalomyopathy	Combined OXPHOS deficiency	8	Homozygous	AR	Yes	*FBXL4*	c.851_852delCT, p.(Pro284Leufs*2)
	Non-consanguineous	3	p	Leigh-like, encephalomyopathy	Combined OXPHOS deficiency	7	Compound heterozygous	AR	Yes	*MTFMT*	c.160G>A, p.(Gly54Ser);c.626C>T, p.(Arg181SerfsX5)
	Consanguineous	1	p	Encephalopathy, lactic acidosis, pulmonary hypertension,	CI deficiency	5	Homozygous	AR	Yes	*NDUFAF4*	c.23G>A, p.(Gly8Asp)
	Consanguineous	2	p	Leigh syndrome, cardiomyopathy	Combined OXPHOS deficiency	5	Homozygous	AR	Yes	*QRSL1* (Kamps et al., 2018)	c.850-3G>A, aberrant splicing of exon 8
	Consanguineous	1	p	Optic atrophy, cerebellar atrophy, hypotonia, increased blood lactate	CIV deficiency	8	Homozygous	AR	Yes	*SLC25A46* (Nguyen et al., 2017)	c.283+3G>T, p.(Ser32Thrfs*4)
	Consanguineous	1	p	Hearing problems, muscle hypotonia, mitochondrial encephalopathy, motor developmental delay, respiratory problems, mtDNA depletion	CI and CIV deficiency	8	Compound heterozygous	AR	No	*RRM2B*	c.142C>T, p.(Gln48*); c.431C>T p.(Thr144Ile)
	Non-consanguineous	2	p	Axonal neuropathy, mild neurodegenerative disorder	Combined OXPHOS deficiency	5	Compound heterozygous	AR	Yes	*COQ7*	c.197T>A, p.(Ile66Asn); c.446A>G, p.(Tyr149Cys)
	Consanguineous	1 (6 miscar.)	p	Leigh-like, dystonia, motor developmental delay	CI deficiency	5	Homozygous	AR	Yes	*NDUFAF5*	c.477A>C, p.(Leu159Phe)
	Consanguineous	1	p	Optic neuropathy, failure to thrive, 3-methylglutaconic aciduria	CI deficiency	5	Homozygous	AR	Yes	*TMEM126A*	c.163C>T, p.(Arg55*)
	Consanguineous	3	p	Microcephaly, neurodegeneration, psychomotor retardation, cerebral visual impairment, hypotonia	CII and CIII deficiency	6	Homozygous	AR	Yes	*PYCR2*	c.796C>T, p.(Arg266*)
	Consanguineous	3	p	Leigh syndrome	Normal, decreased oxygen consumption	7	Homozygous	AR	No	*SLC19A3* (Gerards et al., 2013)	c.20C>A, p.(Ser7*)
	Consanguineous	2	p	Leigh syndrome	Normal, decreased oxygen consumption	7	Homozygous	AR	No	*SLC19A3* (Gerards et al., 2013)	c.20C>A, p.(Ser7*)
	Non-consanguineous	2	p	Psychomotor retardation, white matter degeneration, hypo-myelination, failure to thrive	CII and CIV deficiency	6	X-linked	AR	No	*SLC16A2*	c.427_430+19delGCAGGTGAGTGGCCCCGCACGCC, splice donor site intron 1 deleted
	Consanguineous	1	p	Leigh-like, white matter degeneration, epilepsy, dystonia, visual problems, increased blood lactate	n.d.	7	Homozygous	AR	No	*IER3IP1*	c.128G>T, p.(Gly43Val)
**Autosmal recessive, X-linked, autosomal dominant (non-consanguineous, 1 patient)**	Non-consanguineous	1	a	CPEO, deafness, multiple mtDNA deletions	n.d.	5	Compound heterozygous	AR	Yes	*POLG1*	c.752C>T, p.(Thr251Ile); c.1760C>T, p.(Pro587Leu)
	Non-consanguineous	1	p	Epilepsy, multiple mtDNA deletions	n.d.	5	Homozygous	AR	Yes	*POLG1*	c.1399G>A, p.(Ala467Thr)
	Non-consanguineous	1	p	Leigh syndrome	CI and CIII deficiency	6	Homozygous	AR	Yes	*NDUFS7*	c.364G>A, p.(Val122Met)
	Non-consanguineous	1	p	Cardiomyopathy, cerebellar atrophy	Combined OXPHOS deficiency	6	Compound heterozygous	AR	yes	*MTO1*	c.253G>A, p.(Gly85Arg); c.938G>A, p.(Arg313Gln)
	Non-consanguineous	1	a	Myopathy, ptosis, spinocerebellar ataxia, ragged red fibers	Normal	6	Compound heterozygous	AR	yes	*SPG7*	c.1529C>T, p.(Ala510Val), c.2090A>C, p.(Gln697Pro)
	Non-consanguineous	1	p	Leigh syndrome	CI deficiency	5	Homozygous	AR	yes	*NDUFA12*	c.83dup, p.(Arg29Glnfs*4)
	Non-consanguineous	1	p	SNHL, psychomotor retardation, spasticity, epilepsy	Decreased ATP production	5	Compound heterozygous	AR	yes	*KARS*	c.1732_1744delGGCATTGATCGAG, p.(Gly578Serfs*20); c.683C>T, p.(Pro228Leu)
	Non-consanguineous	1	p	Encephalopathy, white matter degeneration	CI deficiency	6	Homozygous	AR	yes	*NDUFV2*	c.547G>A, p.(Ala183Thr)
	Non-consanguineous	1	p	Leigh-like, microcephaly, mental retardation, CPEO, dystonia	Combined OXPHOS deficiency	6	Compound heterozygous	AR	yes	*MTFMT*	c.626C>T, p.(Ser209Leu); c.994C>T, p.(Arg332*)
	Non-consanguineous	1	p	Leigh-like, small cerebellum, high lactate	n.d	6	Compound heterozygous	AR	yes	*FBXL4* (van Rij et al., 2016)	c.292C>T, p.(Arg98*); c.1303C>T, p.(Arg435*)
	Non-consanguineous	1	p	Myopathy, muscle weakness, visual problems, mental retardation, peripheral neuropathy, mtDNA depletion	n.d.	6	Homozygous	AR	no	*C19orf12*	c.187G>C, p.(Ala63Pro)
	Non-consanguineous	1	p	Exercise intolerance, muscle weakness	CI deficiency	5	Homozygous	AR	yes	*TMEM126B* (Theunissen et al., 2017a)	c.635G>T, p.(Gly212Val)
	Non-consanguineous	1	p	Leigh syndrome, liver failure	CI and CIV deficiency	6	Homozygous	AR	yes	*TRMU*	c.1073_1081dup, p.(Gln358_Val360dup); deletion exon 2-11
	Non-consanguineous	1	p	Mental retardation, ataxia, exercise intolerance, 3-methylglutaconic aciduria	CV deficiency	7	Homozygous	AR	yes	*ATPAF2*	c.281G>C, p.(Trp94Ser)
	Non-consanguineous	1	a	Leigh-like, optic atrophy, exercise intolerance	n.d.	7	Homozygous	AR	yes	*AMACR*	c.154T>C, p.(Ser52Pro)
	Non-consanguineous	1	p	Optic atrophy, muscle weakness, polyneuropathy	combined OXPHOS deficiency	5	Homozygous	AR	yes	*C12ORF65*	c.394C>T, p.(Arg132*)
	Non-consanguneous	1	p	Muscle weakness, cardiomyopathy, high lactate, 3-methylglutaconic aciduria	n.d.	7	X-linked	AR	no	*TAZ*	c.646G>A, p.(Gly216Arg)
	Non-consanguineous	1	p	Cerebellar atrophy, ataxia, mental retardation, white matter abnormalities, axonal peripheral sensorimotor neuropathy	n.d.	5	*de novo*	AD	yes	*MFN2*	c.1058C>T, p.(Ala353Val)
	Non-consanguineous	1	a	Cardiac arrhythmia, CPEO, polyneuropathy, multiple mtDNA deletions	CII and CIV deficiency	5	unknown#	AD	yes	*twinkle (c10orf2)*	c.1087T>C, p.(Trp363Arg)
	Non-consanguineous	1	p	Arthrogryposis, foot deformities, muscle fat depositions, oculocutaneous albinism	CIV deficiency	6	*de novo*, compound heterozygous	AD, AR	no, no	*BICD2, HPS1* (Theunissen et al., 2017b)	c.539A>C, p.(Asp180Ala); c.517C>T, p.(Arg173*); c.1189delC, p.(Gln397Serfs*2)
	Non-consanguineous	1	p	Lung fibrosis, growth retardation, muscle weakness, failure to thrive, hepathopathy	CI deficiency	7	Compound heterozygous	AR	no	*IARS*	c.3377dup, p.(Asn1126Lysfs*9); c.1305G>C, p.(Trp435Cys)
	Non-consanguneous	1	p	CPEO, mitochondrial myopathy, COX negative fibers	CIII deficiency	5	Compound heterozygous	AR	no	*CHRNE*	c.1327delG, p.(Glu443Lysfs*64); c.1429delG, p.(Ala477Profs*30)

*Overview of the gene defects identified by WES in 94 patients, of which 39 had a probable mitochondrial disease. Patients were grouped according to the applied WES-data filtering strategy. AR = autosomal recessive, AD = autosomal dominant, D, dominant; ‘green’ marked genes, genes with a reported mitochondrial function or localization in literature (except for ACY1, ANTXR2, which were part of a multi-genic disease); ‘purple’ marked genes, genes previously related to possibly secondary OXHPHOS deficiencies; ‘red’ marked genes, no mitochondrial localization or function reported; ∧, subclinical symptoms in parent revealed after follow-up investigations; #, no parental DNA available*.

**Table 4:** Disease-causing pathogenic variants identified by WES in group 2, consisting of patients with a possible mitochondrial disorder.

	**Family**	**Patients**	**Symptoms (key features)**	**Biochemistry (muscle or fibroblasts)**	**MDC Score**	**Orientation**	**Inheritance**	**Mito Carta**	**Gene (published by our group)**	**Pathogenic variant**
Autosomal recessive,X-linked(consanguineous, >1 patient)	Consanguineous	2	SNHL, intellectual disability, cerebellar ataxia, peripheral neuropathy, mtDNA copy number increase	Normal	4	Homozygous	AR	Yes	*CLPP Theunissen et al., 2016*	c.21delA, p.(Ala10Profs*117)
	Consanguineous	1	Exercise intolerance, myopathy, ataxia, diabetes mellitus	CI and CIV deficiency	4	Homozygous	AR	Yes	*SLC25A32 (Hellebrekers et al., 2017)*	c.-264_31delinsCTCACAAATGCTCA, deletes start codon
	Consanguineous	1	Limb girdle muscular dystrophy	n.d.	2	Homozygous	AR	No	*CAPN3*	c.1906C>T, p.(Gly636*)
	Consanguineous	1	Spastic diplegia, psychomotor retardation	Normal	2	Homozygous	AR	No	*AP4M1*	c.952C>T, p.(Arg318*)
	Consanguineous	1	Encephalopathy, psychomotor retardation, epilepsy, spastic tetraplegia	CI deficiency	3	Homozygous	AR	No	*ADD3*	c.1100G>A, p.(Gly367Asp)
	Consanguineous	1	Muscle weakness, SNHL, hypotonia, peripheral neuropathy	Normal	4	Homozygous	AR	No	*LMOD3*	c.112delG, p.(Glu38Lysfs*15)
	Non-consanguineous	2	Rhabdomyolysis	Normal	3	Homozygous	AR	No	*LPIN1*	c.1162C>T, p.(Arg388*)
	Consanguineous	2	Encephalopathy, liver failure, psychomotor retardation	Normal	4	Homozygous	AR	No	*NBAS*	c.1556T>A, p.(Val519Gln)
	Non-consanguineous	2	Congenital myopathy, spasticity	Normal	4	Compound heterozygous	AR	No	*SCN4A*	c.2979C>A, p.(Cys993*); c.4949C>T, p.(Pro1650Leu)
	Consanguineous	1	NBIA, microcephaly, epilepsy, psychomotor retardation, cerebellar atrophy	n.d.	3	Compound heterozygous	AR	No	*RELN*	c.4228G>A, p.(Gln1410Lys); c.6866C>T, p.(Thr2289Ile)
Autosmal recessive,X-linked,autosomal dominant(non -consanguineous,1patient)	Non-consanguineous	1	Psychomotor retardation, IBD deficiency, elevated C4-acylcarnitine, PEO	n.d.	4	Homozygous, dominant inherited∧	AR, AD	Yes, yes	*ACAD8, DNA2*	c.289G>A, p.(Gly97Arg); c.2036_2037insAA, p.(His679Glnfs*10)
	Non-consanguineous	1	Cerebellar atrophy, ataxia, dystonia	Normal	2	Compound heterozygous	AR	No	*CWF19L1*	c.37G>C, p.(Asp13His); c.946A>T, p.(Lys316*)
	Non-consanguineous	1	Myopathy, muscle weakness, hypotonia	n.d.	3	*de novo*	AD	No	*ACTA1*	c.16G>A, p.(Gln6Lys)
	Non-consanguineous	1	Epilepsy, hypomyelination, hypotonia, respiratory problems	Normal	4	*de novo*	AD	No	*PURA*	c.802G>T, p.(Gly268*)
	Non-consanguineous	1	Myopathy, epilepsy	Normal	3	*de novo*	AD	No	*DYNC1H1*	c.3364T>C, p.(Ser1122Pro)
	Non-consanguneous	1	Cerebellar ataxia, psychomotor retardation, extrapyramidal syndrome, axonal polyneuropathy	Normal	5	*de novo*	AD	No	*CTNNB1*	c.1511G>A, p.(Trp504*)
	Non-consanguineous	1	Psychomotor retardation, epilepsy, NBIA, retinitis pigmentosa, myopathy	Normal	5	X-linked de novo	D	No	*WDR45*	c.400C>T, p.(Arg134*)
	Non-consanguineous	1	Ataxia, spasticity, psychomotor retardation, cerebellar atrophy, encephalopathy	Normal	4	X-linked de novo (mosaic)	D	No	*CASK*	c.1061T>G, p.(Leu354Arg)

*Overview of the gene defects identified by WES in 94 patients, of which 18 had a possible mitochondrial disease-cause. Patients were grouped according to the applied WES-data filtering strategy. AR, autosomal recessive; AD, autosomal dominant; D, dominant; ‘green’ marked genes, genes with a reported mitochondrial function or localization in literature; ‘red’ marked genes, no mitochondrial localization or function reported*.

Overall, we saw that 31% (13/42) of the disease-causing genes in the mitochondrial patients (group 1) were not included in the MitoCarta database for mitochondrial localized proteins at the time we identified the genetic defect (Table 3). Surprisingly, this included 3 genes with a literature reported function in mitochondrial metabolism, in which pathogenic variants are a well-known cause of mitochondrial disease (*RRM2B, c19orf12, TAZ*). Furthermore, we identified 2 different genes (*SLC19A3* (2x), *SLC16A2*) for which a role in mitochondrial OXPHOS is still unclear. Although a mitochondrial function cannot be excluded, it has been suggested that the OXPHOS deficiencies in the corresponding patients, might be secondary (Niyazov et al., 2016; de Beaurepaire et al., 2018). Furthermore, 5 different genes with no reported function in mitochondrial metabolism (*IER3IP1, IARS, CHRNE, BICD2, HPS1*) have been identified. These genes require further functional testing to reveal a possible “novel” role in mitochondrial functioning or to demonstrate a secondary respiratory deficiency. In the patient with the multi-genic cause (*BICD2* and *HPS1*), an additional, third, gene defect might have been missed. Two additional genes that were lacking in the MitoCarta database were not related to the mitochondrial symptoms of the patients, as these genes encode proteins with no mitochondrial function, but were together with a mitochondrial gene defect part of a complex multi-genic disease phenotype, in which more than a single gene defect is involved (*ACY1, ANTXR2*).

Genes identified in group 1 (MD-patients) were clustered according to their function (Table 5). Most genetic defects were detected in genes related to mitochondrial protein metabolism (protein translation and degradation) and OXPHOS function (ETC subunits, assembly factors, and cofactors). The first group mainly consisted of mitochondrial tRNA synthethases, transferases, and modification enzymes, where the resulting defects in mitochondrial protein synthesis (*AARS2, MTFMT, MTO1, QRSL1, TRMU, C12ORF65*) especially manifested with combined OXPHOS deficiencies (not measured for *KARS*). Among the group of OXPHOS-associated genes, defects in complex I subunits and assembly factors (*NDUFA12, NDUFS7, NDUFV2, TMEM126A, TMEM126B, NDUFAF4, NDUFAF5*) were most prevalent, all resulting in a significant complex I deficiency at the biochemical level. *NDUFS7* resulted in an additional complex III deficiency. Defects in *NDUFS7, NDUFA12* and *NDUFAF5* caused Leigh-syndrome or a Leigh-like-phenotype. The third largest group represented genes involved in mtDNA metabolism, required for mtDNA maintenance, replication and nucleotide metabolism. The *RRM2B* defect resulted in mtDNA depletion, whereas pathogenic variants in the RMM2B interacting protein PYCR2 did not reveal any mtDNA abnormalities. Furthermore, pathogenic variants in *POLG1, twinkle* (*c10orf2*) and *DNA2* displayed multiple mtDNA deletions in muscle. Interestingly, we found the mitochondrial localized, but functionally uncharacterized, *c19orf12* gene to be associated with mtDNA depletion (mtDNA copy-number, normalized to nDNA, was 10–30% of healthy control samples). Among the mitochondrial patients (group 1), we identified pathogenic variants in 5 genes without a reported mitochondrial function or localization, but reported to result in a comparable phenotype as in our patient. 4 genes, functional in different cellular processes, including the transporter proteins SLC19A3 (thiamine) and SLC16A2 (thyroid hormones), tRNA-synthetase IARS, and acetylcholine receptor subunit CHRNE, were likely to underlie the OXPHOS deficiencies measured in these patients, although some of these genes have been related to possible secondary OXPHOS deficiency (SLC19A3 and SLC16A2). In an additional patient, the functionally uncharacterized *IER3IP1* gene was likely to explain a seemingly mitochondrial disease phenotype, yet no OXPHOS measurement was performed in this patient. As indicated in Table 6, in patient's from group 2, 4 mitochondrial gene defects were identified, *SLC25A32, CLPP, ACAD8*, and *DNA2* (multi-genic disease cause).

**Table 5:** Mitochondrial gene functions affected in mitochondrial patients (group 1).

**Gene**	**Function**	**References**
**Mitochondrial substrate metabolism**		
*AMACR*	Alpha-methylacyl-CoA racemase	Mubiru et al., 2004
**Mitochondrial protein metabolism; translation and degradation**		
*AARS2*	alanyl-tRNA synthetase	Götz et al., 2011
*MTFMT (2x)*	Mitochondrial methionyl-tRNA formyltransferase	Takeuchi et al., 1998
*MTO1*	tRNA modification and protein synthesis	Ghezzi et al., 2012
*KARS*	lysyl-tRNA synthetase	Targoff et al., 1993
*QRSL1*	glu-tRNA synthetase	Nagao et al., 2009
*TRMU*	Mitochondrial tRNA modification	Yan and Guan, 2004
*C12ORF65*	Release proteins from ribosomes	Antonicka et al., 2010
*SPG7(2x)*	Part of the m-AAA metalloproteinase complex	Warnecke et al., 2007
**Mitochondrial phospholipid metabolism**		
SERAC1*	Phosphatidylglycerol remodeling	Wortmann et al., 2012
*TAZ*	Cardiolipin remodeling	Acehan et al., 2011
**ETC subunits, assembly factors, cofactors**		
*NDUFA12*	Complex I subunit	Triepels et al., 2000
*NDUFS7*	Complex I subunit	Visch et al., 2004
*NDUFV2*	Complex I subunit	de Coo et al., 1997
*COQ7*	Coenzyme Q biosynthesis	Freyer et al., 2015
*TMEM126B*	Complex I assembly factor	Andrews et al., 2013
*ATPAF2*	Complex V assembly factor	Wang et al., 2001
*NDUFAF5*	Complex I assembly factor	Sugiana et al., 2008
*NDUFAF4*	Complex I assembly factor	Karp et al., 2004
*TMEM126A*	Complex I assembly factor	Wessels et al., 2013
**mtDNA maintenance, replication and nucleotide metabolism**		
*RRM2B*	Ribonucleotide reductase	Bourdon et al., 2007
*FBXL4 (2x)*	Phosphorylation-dependent ubiquitination	Bonnen et al., 2013
*PYCR2*	Pyrroline-5-carboxylate reductase	Kuo et al., 2016
*Twinkle (C10ORF2)*	mtDNA helicase	Spelbrink et al., 2001
*POLG1 (2x)*	mtDNA polymerase	Lestienne, 1987
**Mitochondrial fusion and fission**		
*SLC25A46*	Interacts with mitochondrial fusion machinery	Janer et al., 2016
*MFN2*	Mitochondrial fusion	Santel and Fuller, 2001
**Mitochondrial localization**		
*C19orf12*	Function unclear	Landoure et al., 2013
**No mitochondrial localization or function reported**		
*SLC19A3 (2x)*	Transmembrane thiamine transporter	Vernau et al., 2015
*IER3IP1*	Function unclear	Yiu et al., 2004
*SLC16A2*	Transporter of thyroid hormones	Wrutniak-Cabello et al., 2001
*CHRNE*	Acetylcholine receptor subunit	Witzemann et al., 1996
*IARS*	Isoleucyl-tRNA synthetase	Kopajtich et al., 2016
*BICD2, HPS1(multi-genic)*	dynein-mediated transport, forms lysosomal complex	Martina et al., 2003; Neveling et al., 2013b

Disease causing nuclear genes, identified in patients with a mitochondrial disorder (group 1), clustered according to their function in mitochondrial metabolism. *ACY1 and ANTXR2 were excluded.

**Table 6:** Mitochondrial gene functions affected in patients from group 2.

**Gene**	**Function**	**References**
**Mitochondrial substrate import**		
*SLC25A32*	Mitochondrial folate transporter	Haitina et al., 2006
**Mitochondrial protein metabolism; translation and degradation**		
*CLPP*	Mitochondrial proteolytic complex	Jenkinson et al., 2013
**Mitochondrial substrate metabolism**		
*ACAD8*	Isobutyryl-CoA dehydrogenase	Nguyen et al., 2002
**mtDNA maintenance, replication and nucleotide metabolism**		
*DNA2*	mtDNA replication, mtDNA base-excision repair	Ronchi et al., 2013

*Disease causing nuclear genes were classified according to their function in mitochondrial metabolism*.

## Discussion

We used a next-generation sequencing strategy in a cohort of 86 patients with a likely mitochondrial disease (group 1), as these cases met the criteria for MD, and 31 patients, who did not meet the criteria for MD, but where a mitochondrial defect could possibly cause the disease symptoms. In 68% of the patients we identified a disease causing genetic defect, of which 20% was solved by mtDNA sequencing and 49% by subsequent WES analysis. Compared to conventional Sanger sequencing methods, which only solved 11% (Neveling et al., 2013a), primarily due to limitations on the number of genes being sequenced, this is a major step forward. Selective sequencing of targeted gene panels in two studies, including 1,598 and 1,034 mitochondrial disease genes, resulted in respectively 22% (102 suspected mitochondrial patients) (Lieber et al., 2013) and 24% (42 mitochondrial patients) (Calvo et al., 2012) diagnostic yield. This is less than in our study, which might in part be explained by an incompleteness of the panels (more details below). In a previous next-generation sequencing-based study, which included 113 pediatric patients with suspected mitochondrial disease, screening both the mtDNA and the exome, resulted in a diagnostic yield of 59% (Pronicka et al., 2016). Another study performing WES in 109 pediatric and young adult patients with suspected mitochondrial disease, established a genetic diagnosis in 39% of the patient cohort (after exclusion of mtDNA mutations) (Wortmann et al., 2015). Our pediatric patient group consisted of 90 patients, for which we could establish a genetic diagnosis in 66% (8 mtDNA and 51 nuclear gene mutations). Our higher yield could be caused by a complete analysis of both the mtDNA and the exome, with a strong selection for genetic cases, and follow-up investigations in case of an unknown gene. The relatively high number of patients from consanguineous parents could in part explain the high diagnostic yield, as a genetic cause is highly likely. As consanguineous parents have an increased risk of having children that suffer from multiple genetic diseases, WES data should always be completely analyzed and, preferably, parents should be offered preconception genetic testing. Different from previous studies on mitochondrial cohorts, we have included a more heterogeneous patient group (group 2), in which a relatively small portion is likely to be caused by a mitochondrial gene defect. In conclusion, our data shows that mtDNA sequencing followed by a complete whole exome analysis is the preferred strategy to identify the genetic basis in heterogeneous neuromuscular patients with a likely or possible mitochondrial disease cause.

As the mtDNA genome is relatively small, NGS allows cost effective and efficient testing of many patient samples within a single run, with sufficient sensitivity on blood DNA. Yet, as mtDNA mutations may disappear from blood during life, in cases where an mtDNA disorder is likely, DNA from muscle (or urine for the m.3243A>G) should be tested to prevent missing a diagnosis. Also for prognosis mutation levels in muscle are preferable. Where traditionally several complementary methods had to be used for detection and quantification of mtDNA mutations (e.g., RFLP, ARMS-qPCR, sanger sequencing, Mito-CHIP, Southern blot analysis), NGS can be used as a single method for identifying point mutations and indels, large deletion breakpoints and quantifying heteroplasmy levels of point mutations and small indels with high sensitivity and specificity (Tang and Huang, 2010). Only to accurately measure heteroplasmy levels of large mtDNA deletions and mtDNA depletions, additional qPCR based quantification is required. NGS of the mtDNA solved 20% of our patient cohort (all mitochondrial patients), mainly, but not only, consisting of patients with early disease onset (77% pediatric patients). In the pure pediatric subgroup with mitochondrial disease (group 1), mtDNA mutations accounted for 19% (8/43) of the genetic defects, which is in line with earlier reports that estimated involvement of a mtDNA defect in <20% of the pediatric mitochondrial patients (Schaefer et al., 2008). In our adult subgroup with mitochondrial disease (group 1), mtDNA defects accounted for 75% (12/16), which is comparable to the previously estimated 70–75% (Schaefer et al., 2008; Gorman et al., 2015). As shown in Table 1, most mtDNA patients carried LHON and MIDD/MELAS causing mutations, which was also likely based on the clinical presentation. Both MIDD and MELAS symptoms were caused by the m.3243A>G mutation, in line with what has been reported before (de Wit et al., 2012). Yet, as indicated by the heteroplasmy levels (24–41% in MELAS and 7–40% in MIDD), variation in symptom manifestations among these patients could not solely be explained from the differences in heteroplasmy level in blood, complicating prognostic predictions.

Using WES, we identified a disease-causing variant in an additional 49% of the patient cohort, where we applied a variant selection strategy fitting the presumed genetic mode of disease inheritance. In a group with likely inherited disease (consanguineous and/or multiple patients), filtering for recessive pathogenic variants solved 68% of the patients. A group of single patients from non-consanguineous parents was also screened for heterozygous variants in known (OMIM) AD disease genes, which resulted in an overall genetic diagnosis in 56% of the patients, of which 15% of the identified variants were *de novo*. To reduce the huge amount of potential dominant pathogenic variants, we have restricted our heterozygous variant selection to OMIM-reported dominant disease genes, and therefore might have missed some *de novo* cases. This could be overcome by WES trio-analysis. Additionally, the higher likelihood of an inherited genetic cause, the availability of homozygosity mapping data and of exome data of affected siblings might have contributed to a higher diagnostic yield in the first group. Our strategy did not result in large differences in diagnostic yield between patients from group 1 and patients from group 2. In an additional 7% of the WES cohort we found a genetic variant (variant of unknown significance) with a clear lead to the patient's phenotype (data not shown). For these cases, further laboratory testing or patient screening should reveal which genes can be classified as disease causing.

In 82% (32/39) of the likely mitochondrial patients (group 1), symptoms were caused by a gene with a reported mitochondrial function or localization. As expected, this was significantly lower in patient group 2 [17% (3/18)]. Our results showed that in patients with mitochondrial disease, most genetic defects were associated with mitochondrial protein metabolism and OXPHOS function, where a majority of the OXPHOS defects were found in complex I genes. In line with previously reported complex I deficient patients, we found that defects in *NDUFS7, NDUFA12*, and *NDUFAF5* caused Leigh-syndrome or a Leigh-like-phenotype (Rahman and Thorburn, 1993). Although *NDUFV2* defects have also been reported in association with Leigh syndrome (Cameron et al., 2015), our patient exclusively expressed symptoms of white matter degeneration (encephalopathy). Also, we identified *TMEM126A* as an underlying cause of isolated complex I deficiency, therewith supporting co-migration studies that characterized TMEM126A as an early assembly factor of complex I (Wessels et al., 2013). Patients with mtDNA abnormalities carried pathogenic variants in *RRM2B, twinkle* (*c10orf2*) and *DNA2*, which were all functionally related to mtDNA maintenance, and presented as the third largest group. Interestingly, we also found pathogenic variants in the *c19orf12* gene in a patient with an mtDNA depletion. The link between this gene and the mtDNA depletion remains to be established.

In 6 mitochondrial patients (group 1), we identified a causal gene for which no direct mitochondrial function or localization has been reported. This included 6 genes involved in different cellular processes (*SLC19A3, SLC16A2, IARS, CHRNE, BICD2*, and *HPS1*). In two families with classical Leigh-syndrome we identified a defect in the thiamine transporter SLC19A3, where despite a decrease in mitochondrial oxygen consumption, complex activities seemed normal in muscle, and skin tissue (Gerards et al., 2013). Thiamine-diphosphate, the active form of thiamine, has been reported as an essential cofactor for several mitochondrial enzymes, including the pyruvate dehydrogenase complex (Ortigoza-Escobar et al., 2014). A second transmembrane transporter defect was found in the thyroid hormone (T3/T4) transporter SLC16A2, in a patient with Allan-Herndon Dudley syndrome, which resulted in a CII and CIV deficiency, where triiodothyronine (T3) has been suggested as an important regulator of mitochondrial activity (Wrutniak-Cabello et al., 2001). However, a role for SLC19A3, but also SLC16A2 as mitochondrial genes is under debate, as defects in these genes have been suggested to cause possible secondary respiratory chain deficiency (Niyazov et al., 2016; de Beaurepaire et al., 2018). In such case, patients might possibly get a false positive classification as mitochondrial (group 1) based on biochemical criteria Still, concerning SLC19A3, either being primary or secondary, this gene should be tested in patients with Leigh syndrome, and should be in all MD panels, because it is a treatable condition, even though, having a secondary effect, it may not have been included in the MitoCarta database. Furthermore, we found a pathogenic variant in the cytoplasmic located aminoacyl-tRNA synthetase IARS to cause complex I deficiency in skin and muscle tissue of a patient with a multi-system disorder, and a defect in the acetylcholine receptor subunit epsilon CHRNE associated with CII deficiency, COX negative muscle fibers and abnormally shaped muscle mitochondria in CPEO with myopathy. Also we measured a complex IV deficiency in a patient with a multi-genic cause, where either the identified *BICD2* or *HPS1* defect causes the deficiency, or a possible third defect was missed. In another patient, who was classified as mitochondrial based on the classical mitochondrial disease symptoms involved, the functionally uncharacterized *IER3IP1* gene was identified. Although, no OXPHOS measurements were performed in this patient, knockdown of IER3IP1 was reported to affect mitochondrial function by decreasing the mitochondrial membrane potential and increasing cytochrome C release (Sun and Ren, 2017). The genetic defects in these patients need further functional characterization to prove or exclude a mitochondrial function, the involvement of secondary respiratory chain deficiencies, or perhaps the presence of an additional gene defect that has been missed by WES.

31% of the disease-causing genes identified in our mitochondrial patients (group 1) were not included in the MitoCarta database at the moment of identification, and could therefore be missed with panel-based sequencing approaches that rely on the MitoCarta database. In addition to the 5 genes described above, this also involved some genes with a reported function in mitochondrial metabolism, indicating that it can be difficult to keep databases or panels up to date with recent discoveries. Also, the contribution of non-mitochondrial disease genes to complex multi-genic mitochondrial disease might be missed when applying targeted sequencing methods, as illustrated by two of the mitochondrial patient cases, in which defects in more than a single gene were involved. Our data shows that WES is the most suitable approach to characterize the causative gene defect in both groups, patients with most likely a mitochondrial disease, as according to the consensus criteria for MD (group 1), and patients in who a mitochondrial gene defect could possibly be involved (group 2). With respect to group 1, we show mitochondrial gene panels are still not complete, and that a strict preselection based on the consensus criteria for MD could result in the missing of mitochondrial gene defects (*SLC25A32, CLPP, ACAD8, DNA2*). Also in group 2 WES will be the preferred method, because of the genetic heterogeneity, targeted approaches can easily result in inefficient and sequential use of different gene panels, and still miss the complete picture in multi-genic disease. WES is a comprehensive and unbiased approach to establish a genetic diagnosis in heterogeneous mitochondrial disease, able to resolve complex multi-genic disease manifestations. Obviously, a disadvantage of WES in diagnostics settings will be the identification of novel, non-reported gene defects that often require extensive experimental setups to validate a possible role in mitochondrial disease. Embedding within or contact with a specialized research group should therefore be preferable. Identification of the genetic defect is not only important for diagnostics, but also for therapeutic interventions. Patients with *SLC25A32, SLC19A3*, and *TMEM126B* defects showed improvement upon treatment with respectively riboflavin, biotin/thiamine and high fat-diet (Gerards et al., 2013; Hellebrekers et al., 2017; Theunissen et al., 2017a). Yet, despite the significant improvements in the genetic diagnosis of mitochondrial disorders, the development of novel therapies is lagging behind.

## Author contributions

TT, MN, RK, JV, DH, and MG were responsible for analysis and interpretation of patient WES data, and validation of the variants. RG, AH, and CC were involved in the handling of patient DNA and WES preparation. BdK, AS, and RS were responsible for the bioinformatics pipeline. KS performed biochemical measures on patient material. EM, SF, YH-H, MdV, IdC, and SS was responsible for patient contact and clinical analysis and diagnosis. IdC, DH, and HS were responsible for intellectual content and study setup.

### Conflict of interest statement

The authors declare that the research was conducted in the absence of any commercial or financial relationships that could be construed as a potential conflict of interest. The reviewer CLA declared a past co-authorship with the authors SS and HS to the handling Editor.
