# Supplementary material for: Whole Exome Sequencing Is the Preferred Strategy to Identify the Genetic Defect in Patients With a Probable or Possible Mitochondrial Cause
Source: Front Genet. 2018 Oct 12;9:400. doi: 10.3389/fgene.2018.00400 (PMC6194163; doi:10.3389/fgene.2018.00400)
Supplement: Supplementary file 1 [file Data_Sheet_1.pdf]

Supplementary data:

Table S1: Quantification of heteroplasmic mtDNA point mutations using RFLP and NGS. Percentages of the heteroplasmic point mutations m.3243A>G (3 samples), m.8993T>G (1 sample) and variant m.9363A>G (1 sample) determined by NGS appeared to be in good agreement with the mtDNA heteroplasmy levels previously determined by RFLP.

		RFLP	NGS		
**sample**	**mutation**	**% heteroplasmy**	**coverage**	**genotype**	**% heteroplasmy**
3	m.9363A>G	7%	13175	A>AG	10,3
4	m.8993T>G	97%	16644	T>TG	99,2
5	m.3243A>G	17%	10890	A>AG	22,4
6	m.3243A>G	16%	14556	A>AG	20,8
7	m.3243A>G	53%	7565	A>AG	51,7

Table S2: Comparison of theoretical heteroplasmy and heteroplasmy detected by NGS in 2 mixed mtDNA samples. For additional prove of reliable heteroplasmy quantification, a range of heteroplasmy levels was mimicked by mixing 2 samples at different proportions. The heteroplasmy levels of the variants determined by NGS show excellent correlation with the theoretical levels. In addition, the detection limit of heteroplasmy appeared to be about 1%. Data are shown for a specific mtDNA variant present in 1 of the 2 samples but are representative for all variants.

**expected heteroplasmy (%)**	100,0%	99%	97,5%	95%	90%	75%	25%	10%	5%	2,5%	1%	0%
**observed heteroplasmy (%)**	99,2	98,4	97,1	94,8	90,6	75,6	24,1	9,5	4,99	2,67	1,29	0,34

Table S3: Analyzing small insertions and deletions with NGS, as previously detected by Sanger. To validate detection of small mtDNA insertions and deletions, NGS was performed for samples with a known homoplasmic 1bp insertion (3 samples) or deletion (4 samples). All these variants were detected by NGS at nearly homoplasmic levels. All variants detected with Sanger are also called by NGS.

sample	Sanger	coverage	genotype	% heteroplasmy
8	m.2232dupA	8598	m.2232dupA	92,5
9	m.3229_3230insA	16484	m.3229_3230insA	93,3
10	m.12306dupA	9568	m.12306dupA	92,1
11	m.2356delA	8480	m.2356delA	96,8
12	m.2395delA	8268	m.2395delA	97,4
13	m.4317delA	11004	m.4317delA	94,5
14	m.15944delT	12360	m.15944delT	95,9

Table S4. Detection of large mtDNA deletions by NGS. Deletion sizes and breakpoints are compared between NGS and PCR/Southern Blotting. In case a single large mtDNA deletion is detected by initial LR-PCR agarose gel analysis, further mtDNA deletion analysis is performed by NGS. To validation large deletion detection by NGS, five patient samples with known large mtDNA deletions were analysed by NGS. All single mtDNA deletions are clearly identified by a decreased read depth, with correct deletion sizes

	Soutern Blot / PCR		NGS	
**sample**	**deletion size**	**breakpoints**	**deletion size**	**breakpoints**
15	3543	9515:13059	3543	9515:13059
16	3914	7969:11884	3914	7969:11884
17	4977	8482:13460	4977	8482:13460
18	5408	8927:14336	5408	8927:14336
19	3438	11589:15028	3438	11589:15028

Table S5: Comparison of MitoChip with NGS A) All homoplasmic mtDNA variants previously detected by MitoChip in two samples were confirmed by NGS. No additional homoplasmic or nearly homoplasmic variants were found. **B)** . Heteroplasmic NGS calls (heteroplasmy cut-off 5%), excluding the D-loop and known pathogenic mutations. Because NGS provides heteroplasmy levels for each position of the mtDNA, the number of heteroplasmic variant calls depends on the applied heteroplasmy cut-off. Using a 5% heteroplasmy cut-off, the number of additional heteroplasmic variants called in the 2 samples (not previously detected with MitoChip) was 5 and 4, respectively. These additional calls were either due to a repeat in the non-coding part of the mtDNA, or appeared to be present in all tested NGS samples at low heteroplasmy levels (2%-8%), suggesting these are artificial 'background' calls (although the true presence of these variants at low heteroplasmy levels in all samples cannot be completely ruled out).

A

sample 1

MitoChip		NGS		
**position**	**genotype**	**coverage**	**genotype**	**heteroplasmy**
709	G>AA	9497	G>GA	99,3
750	A>GG	10857	A>AG	99,2
1438	A>GG	16464	A>AG	99,3
2706	A>GG	12895	A>AG	99,5
3537	A>GG	4224	A>AG	99,0
3866	T>CC	14830	T>TC	99,5
4086	C>TT	15925	C>CT	99,6
4769	A>GG	16973	A>AG	99,1
6875	C>TT	16254	C>CT	99,3
6960	C>TT	15927	C>CT	99,7
7028	C>TT	15163	C>CT	99,3
8584	G>AA	14526	G>GA	99,4
8860	A>GG	17977	A>AG	99,2
9950	T>CC	11508	T>TC	99,4
10398	A>GG	8998	A>AG	99,5

11465	T>CC	17318	T>TC	99,7
11719	G>AA	20863	G>GA	99,4
14766	C>TT	15442	C>CT	99,0
14962	C>TT	18757	C>CT	99,4
15235	A>GG	20567	A>AG	99,4
15326	A>GG	19803	A>AG	98,5

sample 2

**MitoChip**		**NGS**		
**position**	**genotype**	**coverage**	**genotype**	**heteroplasmy**
750	A>GG	6640	A>AG	99,4
769	G>AA	7137	G>GA	99,6
1018	G>AA	8269	G>GA	99,5
1438	A>GG	8714	A>AG	99,3
2416	T>CC	7853	T>TC	99,2
2706	A>GG	7671	A>AG	99,5
2789	C>TT	7785	C>CT	99,4
3594	C>TT	4626	C>CT	99,0
4104	A>GG	13838	A>AG	98,9
4769	A>GG	13280	A>AG	99,1
5581	A>GG	5200	A>AG	98,9
7028	C>TT	10237	C>CT	99,1
7175	T>CC	8579	T>TC	99,7
7256	C>TT	7359	C>CT	99,7
7274	C>TT	7168	C>CT	99,4
7521	G>AA	7302	G>GA	98,8
7771	A>GG	9851	A>AG	98,9
8206	G>AA	8805	G>GA	99,5
8701	A>GG	13283	A>AG	98,9
8860	A>GG	16255	A>AG	98,9
9221	A>GG	16804	A>AG	99,1
9540	T>CC	7309	T>TC	99,3
10115	T>CC	4760	T>TC	99,4
10398	A>GG	5555	A>AG	99,2
10873	T>CC	6879	T>TC	99,3
11719	G>AA	18426	G>GA	99,0
11914	G>AA	13714	G>GA	99,3
11944	T>CC	12736	T>TC	99,6
12693	A>GG	10669	A>AG	99,3
12705	C>TT	10460	C>CT	99,4
13590	G>AA	8704	G>GA	99,5
13650	C>TT	8160	C>CT	99,2
13803	A>GG	8420	A>AG	97,1
14566	A>GT	11627	A>AG	98,7
14766	C>TT	11836	C>CT	99,0
15301	G>AA	16001	G>GA	99,2

15326	A>GG	15606	A>AG	98,6
15784	T>CC	13767	T>TC	99,5

B

sample1

**NGS**			
**position**	**coverage**	**genotype**	**heteroplasmy**
8275	7535	C>CA	8,3
8276	7515	C>CT	11,1
8277	7485	T>TA	12,1
8278	7471	C>CG	13,0
8279	7448	T>TC	13,8

sample 2

**NGS**			
**position**	**coverage**	**genotype**	**heteroplasmy**
3572	4108	T>TC	5,3
5467	3918	C>CA	5,3
5471	3854	G>GT	5,6
5477	3961	C>CT	5,2
